# Endogenous amyloidogenesis in long-term rat hippocampal cell cultures

**DOI:** 10.1186/1471-2202-12-38

**Published:** 2011-05-10

**Authors:** Sarah J Bertrand, Marina V Aksenova, Micheal Y Aksenov, Charles F Mactutus, Rosemarie M Booze

**Affiliations:** 1 University of South Carolina, Program in Behavioral Neuroscience, Department of Psychology, Columbia, SC 29208, USA

**Keywords:** Cell Culture, Amyloid Peptide, Neurodegeneration/Aging

## Abstract

**Background:**

Long-term primary neuronal cultures are a useful tool for the investigation of biochemical processes associated with neuronal senescence. Improvements in available technology make it possible to observe maturation of neural cells isolated from different regions of the rodent brain over a prolonged period *in vitro*. Existing experimental evidence suggests that cellular aging occurs in mature, long-term, primary neuronal cell cultures. However, detailed studies of neuronal development *in vitro *are needed to demonstrate the validity of long-term cell culture-based models for investigation of the biochemical mechanisms of *in vitro *neuronal development and senescence.

**Results:**

In the current study, neuron-enriched hippocampal cell cultures were used to analyze the differentiation and degeneration of hippocampal neurons over a two month time period. The expression of different neuronal and astroglial biomarkers was used to determine the cytochemical characteristics of hippocampal cells in long-term cultures of varying ages. It was observed that the expression of the intermediate filament nestin was absent from cultures older than 21 days in vitro (DIV), and the expression of neuronal or astrocytic markers appeared to replace nestin. Additionally, morphological evaluations of neuronal integrity and Hoescht staining were used to assess the cellular conditions in the process of hippocampal culture development and aging. It was found that there was an increase in endogenous production of Aβ_1-42 _and an increase in the accumulation of Congo Red-binding amyloidal aggregates associated with the aging of neurons in primary culture. *In vitro *changes in the morphology of co-existing astrocytes and cell culture age-dependent degeneration of neurodendritic network resemble features of *in vivo *brain aging at the cellular level.

**Conclusion:**

In conclusion, this study suggests that long-term primary CNS culture is a viable model for the study of basic mechanisms and effective methods to decelerate the process of neuronal senescence.

## Background

As a result of the advances in isolation and culturing of fetal and adult central nervous system (CNS) cells achieved during the last two decades [1,2], primary neuronal cell culture has become a powerful tool for isolating cellular and molecular mechanisms of neuronal development and death [1,3,4]. Despite the routine success at long-term culturing of primary rodent neurons [5-9], the application of this potentially very useful approach for modeling of neuronal cell aging remains limited. The standard protocols that allow prolonged CNS cell culturing (including culturing of hippocampal neurons) are now widely available [10,2], however, systematic studies of the differentiation state, cytochemical, and morphological characteristics of brain cells remaining viable over long term *in vitro *are needed.

Although *in vivo *animal models can be used to reveal many important aspects of neuronal development, dysfunction, and degeneration, the inherent complexity of nervous tissue often obscures the overall understanding of molecular, biochemical, and structural observations. The *in vitro *system lacks an intact tissue environment; nevertheless, the presence of a homogenous cell population allows us to identify specific mechanisms underlying the chemical and morphological changes seen *in vivo *[6]. Using long term primary cell culture to decipher the underlying development of the cells of the hippocampus allows examination of where the initial dysfunction takes place that leads to some of the symptoms of neurodegeneration. Understanding what a mature neuron looks like, and when it matures in culture, will help facilitate the development of more efficient *in vitro *testing.

In the current study we used the lack of nestin immunoreactivity, as well as various morphological categorizations, to classify our cultures as mature. Nestin is a type VI neurofilament expressed in the developing CNS, specifically in progenitor cells undergoing differentiation [11-13]. Specific mature neuronal and astrocytic markers, MAP-2 and GFAP, have been found to be co-expressed with nestin in early, primary, neuronal cell cultures [14,11]. The co-localization of these markers and nestin indicate the event of differentiation. Conversely, there should be little to no expression of the intermediate fiber marker nestin in mature primary neuronal cultures [14].

Increased amyloid-beta peptide (Aβ) formation is a common accompaniment of brain aging [15-17]. Accumulation of abnormal protein aggregates, including the misfolded Aβ protein, is the manifestation of progressively deteriorating capacity of aging biological systems to withstand extrinsic and intrinsic hazards [18-20]. Our current study was designed to investigate the possibility of endogenous amyloid beta peptide formation and accumulation of misfolded protein aggregates in hippocampal neurons during their development and maturation *in vitro*.

Aβ peptides arise from the cleavage of amyloid precursor protein (APP) [21]. β-and γ-secretases cleave the unprocessed APP in succession, resulting in the formation of Aβ peptide [15,22,16,23]. Numerous studies have used rodent primary hippocampal cell cultures as an experimental model of normal biogenesis of Aβ [24,13]. Rat hippocampal cells in primary cultures express APP_770_, APP_751_, and APP_695 _[24,25] and the amyloidogenic route of APP processing resulting in the endogenous Aβ_1-40 _or Aβ_1-42 _generation may naturally occur in primary neuronal cultures [26].

The Aβ monomers released from cells transform into β-pleated sheet aggregates to initiate the process of neuronal degeneration associated with the decline in metabolic activity and oxidative damage of cultured hippocampal neurons [27,28]. It was reported that the rodent Aβ is less likely to form large fibrillary structures due to three amino acid substitutions compared to the human sequence. Nevertheless, neurotoxic and pro-oxidant properties of the rat variety of Aβ 1-42 have proven to be the same as human [29]. Most amyloid peptides interact strongly with cell membranes and this interaction is enhanced by conditions which favor β-sheet formation. The excessive release of Aβ from cultured rat hippocampal neurons can result in the increase of amyloid-binding dye fluorescence in the culture medium and Aβ-mediated toxicity [30].

Although increased Aβ formation and the production of aggregates have been observed accompanying the process of neuronal aging [17,18], low levels of Aβ derived from the natural processing of APP isoforms have been found both *in vivo *and *in vitro *[17,22]. Normal physiologic levels of Aβ are thought to be important in regulating neurotransmission [15,16,23,24], consequently many researchers suggest that Aβ should only be regarded as toxic when the production and degradation are imbalanced [15,16].

Overall, the purpose of this study is to investigate the usefulness of long term primary hippocampal cell culture for understanding the link between endogenous Aβ production, differentiation and maturation *in vitro*. In order to discern this link we aim to identify criteria by which a culture can be classified as mature. These classifications can help us properly assess the endogenous levels of Aβ and the accumulation of misfolded Aβ proteins into aggregates as physiological, and not pathological, processes of aging. The ability to classify the maturity of neurons will also strengthen the cell culture model as a whole, giving researchers the ability to more definitively speak about influences on the aging nervous system.

## Results

### The long-term hippocampal cell culture maturation and aging

**Figure 1:**
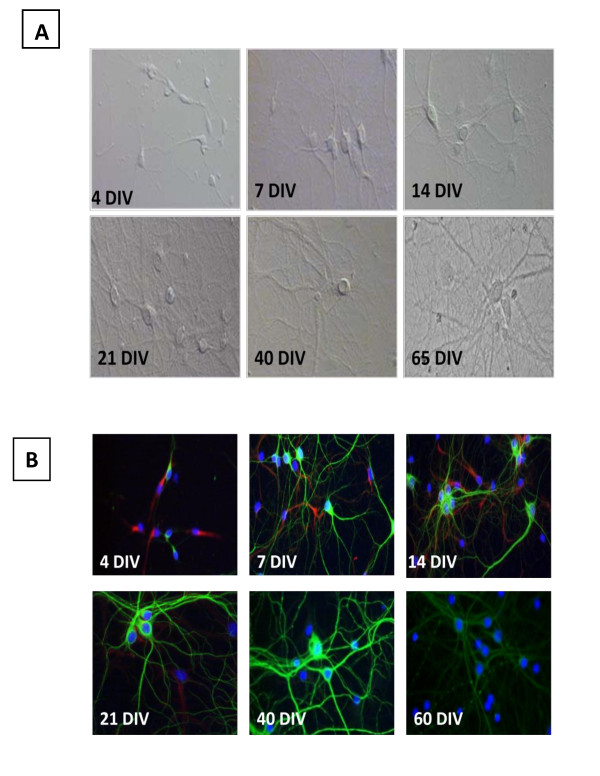
**Rat hippocampal neurons show distinct morphology (MAP-2; green) at different time points (A) and stop expressing the intermediate filament VI, nestin (red), over time (B)**. Cell bodies are stained with Hoerscht. **A**. Young (4 DIV) cells display small soma and short, thin neurites. As time passes the somas become larger and more defined, while neurites elongate (7 - 40 DIV). Once mature (21-40 DIV), the network takes on a progressively more intricate and bundled appearance. At 65 DIV, excessive bundling is present. **B**. The expression of nestin degrades over time (4 DIV- 21 DIV). MAP-2 expression appears to gain intensity up to 40 DIV. At 60 DIV, the MAP-2 expression becomes faded, and takes on a "beaded" appearance on some neurites.

Primary embryonic hippocampal culture preparations used in this study are classified as moderate-high density (160-180 cells/mm^2^). Soon after the adherence to the surface of the cell culture plate, viable hippocampal cells began expressing marker proteins that indicate their commitment to either the neuronal or astrocytic differentiation. The representative DIC microscopy images in figure 1A illustrate that the cells were initially small with no, or a very small number of, neuritic outgrowths at 4 DIV, and later form a visible network by 14 DIV. Extensive networking of neurites and the defined appearance of pyramidal cells at DIV 21-35, indicated this was a stage of maturity for the cell culture. Aging hippocampal cells (DIV 40-65) appeared to have progressively less defined connections with excessive bundling of neurites and larger, more defined cell bodies.

Consistent with the DIC imaging of developing neural networks were images of Nestin/MAP-2 immunofluorescence from the long-term hippocampal cultures of different ages (Figure 1B). In 7-14 DIV hippocampal cells nestin immunoreactivity was generally localized within the axonal growth cones. Neuronal maturation associated with extensive dendritic arborization was characterized by a gradual decline of the expression of this immature neurofilament protein and the increase of MAP-2 expression. As shown in figure 1B, after 14 DIV the majority of the neuronal population in the long-term hippocampal cell culture became predominantly MAP-2 expressing principle pyramidal neurons. Nevertheless, nestin immunoreactivity could be detected in some cells until DIV 21. Hippocampal neuronal cells expressing nestin along with MAP-2 were typically found in cell clusters at this age (21 DIV).

Observations of the mature hippocampal primary cultures during their long-term maintenance clearly showed that the stationary phase, during which cultured cells showed no noticeable changes in morphology, may continue until DIV 40. Signs of neuritic network deterioration began to occur after 40 DIV. Immunoreactivity of MAP-2 in these aged hippocampal cell cultures also revealed varicosities along the processes, giving them a 'beaded' appearance, as shown in figure 1B at 60 DIV.

**Figure 2:**
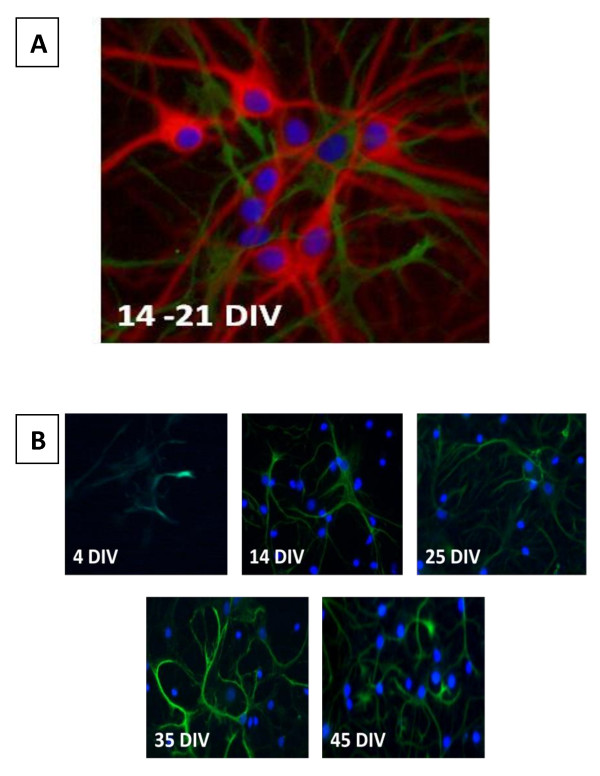
**Astrocytic growth consists of arborization rather than division**. **A**. A representative image of MAP (red) and GFAP (green) expression. Cell nuclei are stained with Hoerscht (blue). Although there are multiple cell bodies present, only one is shown to be expressing GFAP. **B**. At earlier time points, GFAP expression is limited due to the culture medium which does not support astrocytic growth. Although there appears to be an increase in GFAP expression as cultures age, cell bodies associated with GFAP expression remain at similar levels, but there is an increase in arborization.

Primary rat fetal hippocampal cultures were neuron-enriched throughout long-term maintenance. The fully mature hippocampal cell culture (21-35DIV) contained 15 ± 5% GFAP positive cells. The number of astrocytes remained stable throughout the cell culture aging period and did not exceed 20% even in very old (65 DIV) cultures (data not shown). However, the astrocytic cell morphology and GFAP expression changed with the aging of the cell culture age (Figure 2). In long-term hippocampal cultures of 45-60 DIV, astrocytes exhibited very intensive GFAP immunostaining and displayed an extensively branched network of processes (Figure 2B). GFAP expressing cells had thicker processes relative to MAP-2 expressing cells, and their nuclei appeared to be flat and more oblong than MAP-2 expressing cells.

**Figure 3:**
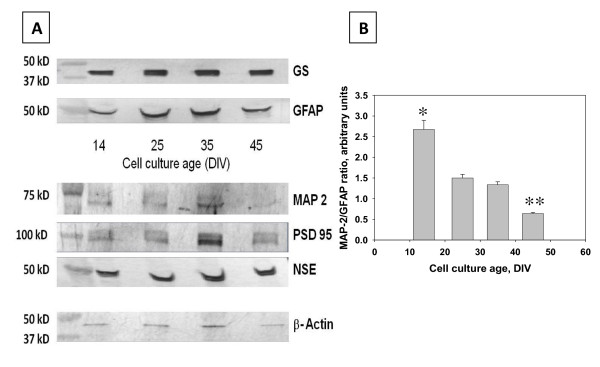
**MAP-2 and GFAP expression changes over time.** A. A comparison of Western blots of specific cell proteins and ubiquitous proteins in both astrocytes and neurons. There is a steady expression of both GS and GFAP throughout the life of the culture, indicating that astrocytes do not over take the dish. MAP-2 and PSD 95 expression increases until DIV 35, at DIV 45 there is a marked drop in the expression of these proteins. This may be due to the fragmentation of neurites. The steady expression of NSE indicates a steady cell population. The steady expression of β-actin indicates that the cell population remains unchanged throughout the life cycle of the cell. **B**. The ratio between MAP-2 and GFAP decreases over time, supporting the western blots for MAP-2 and GFAP. At later time points, although there is an unchanged presence of GFAP, there is a decreased expression of MAP-2.

Results of immunoblotting analyses of neuronal (MAP-2, NSE) and astrocytic (GFAP, GS) marker proteins in hippocampal cell lysates (Figure 3A, B) were consistent with the results of the immunofluorescent microscopy and neuronal/astrocytic cell counting. The MAP-2/GFAP immunoreactivity ratio significantly (P = 0.002 < 0.05) decreased from 2.70 ± 0.22 to 1.50 ± 0.08 between 14 and 25 DIV when hippocampal cell cultures reached the peak of their development. No significant changes in the MAP-2/GFAP ratio have been found in fully mature hippocampal cultures between 25 DIV and 35 DIV (1.50 ± 0.08 and 1.34 ± 0.07; P = 0.191 > 0.05). The end of the stationary phase (35 DIV) was marked by the significant decrease of the MAP-2/GFAP ratio at 45 DIV (0.64 ± 0.03; P = 0.0001 < 0.05), which was attributed to the sharp drop in MAP-2 immunoreactivity but not to the increase in GFAP immunoreactivity. The observed changes in levels of immunoreactive MAP-2 corresponded well with the results of anti-MAP-2 immunofluorescent microscopy, revealing signs of neuronal dendritic network degeneration at this time point (40-50 DIV). Consistently, as shown by the images of the Western blots in Figure 3, the intensity of protein bands corresponding to synaptodendritic markers MAP-2 and PSD 95 exhibited pronounced decreases between 35 DIV and 45 DIV compared to NSE, GS and β-actin.

### Amyloid beta -peptide release and deposition of β-pleated sheet protein aggregates

**Figure 4:**
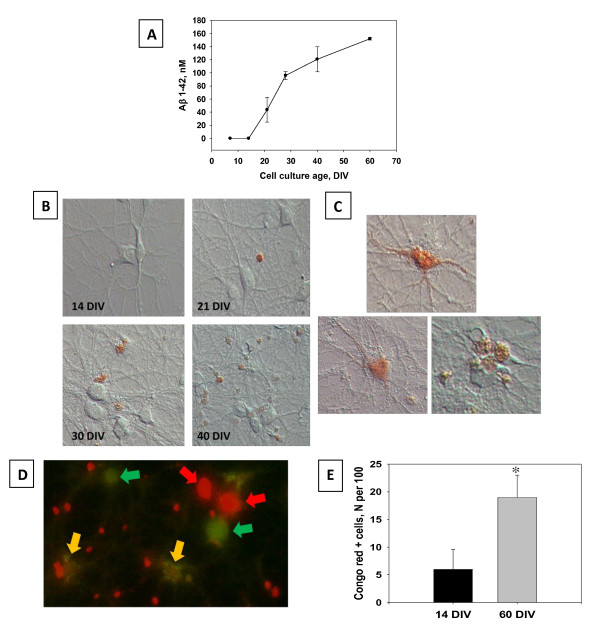
**Long term primary hippocampal cell cultures naturally release Aβ1-42 in increasing amounts over time**. Aβ1-42 is also seen to form an increasing amount of aggregates over time, and is present in live cells. **A**. Aβ1-42 immunoreactivity in the CM samples collected from individual cultures (n = 4 per time point) was measured by a direct ELISA. There was a marked increase in Aβ release beginning at DIV 21. Data are presented as mean values ± SEM. Cell viability in hippocampal cell cultures was determined using Live/Dead assay. **B**. Live cells stained with Congo red dye. Staining with Congo Red is a classical method of the detection of amyloids. Congo red exhibits very low non-specific binding. Congo Red positive staining increases as cells age, beginning at DIV 21 and increasing until DIV 40. **C**. Congo red staining can also be observed in what appears to be the inside of cells, as well as the network. **D**. Congo red (red) can also be observed in live cells at DIV 60. Live cells are marked with calcein (green), and an overlap of orange indicates an overlap of markers. Other Congo red marked cells may be dead at this time point. **E**. Counts of Congo red positive cells significantly (P < 0.05) increase over time.

The presence of specific Aβ 1-42 immunofluorescence in cultured hippocampal neurons indicates the possibility of endogenous Aβ production. Only negligible levels of extracellular Aβ 1-42 immunoreactivity were detected by direct ELISA in the cell culture medium at DIV 7 and DIV 14 (Figure 4A). The progressive accumulation of Aβ 1-42 in the growth medium was starting in the stationary phase of cell culture development (21-35 DIV) and peaked at 120-150 nM during the period of *in vitro *aging between 40 DIV and 60 DIV (Figure 4A).

In this study, the long-term maintenance of hippocampal cell cultures continued until 65 DIV. Live-dead staining was routinely performed to determine cell viability. Double labeling with calcein AM and Congo Red revealed the presence of cells with membrane integrity and the capability to retain the fluorescent product of the calcein AM hydrolysis fairly long into culturing (65 DIV; Figure 4D).

Results of the Congo Red staining of long-term hippocampal cultures at different stages of their development are shown in Figure 4B. Congo Red staining of cell bodies or processes was rarely observed in hippocampal cell cultures until 21 DIV. However, after 35 DIV the Congo Red stained amyloid-like depositions were frequently found in association with the dendritic network and in the cell body (Figure 4C). In cultures older than 40 days, increasing numbers of cells with Congo Red-positive somata were observed (Figure 4E). Total number of cells containing Congo-red positive amyloidal aggregates was much higher in older cells (60 DIV; Figure 4E). Co-labeling with calcein AM and Congo red detected many calcein AM-positive cells some with the presence of Congo Red, as shown by the yellow arrows in Figure 4D.

## Discussion

The technology of CNS cell culturing continues to evolve, bringing forward novel perspectives to advance the understanding of basic principles of neural functioning by using isolated neural cells in a controlled environment. In this study we used the techniques for culturing rat fetal hippocampal neurons developed by Brewer and co-authors [1-3,7,31]. When this method is used, isolated neurons remain viable for long periods of time without the need for astroglial feeder co-cultures [10]. Our own experience [5] and reports from other investigators [7,8,6,32], have documented the successful maintenance of viable cultures containing primary rodent fetal neurons for 60-70 days or longer. The ability to maintain cell cultures for extended periods of time allows further understanding of the fundamental processes of development and aging within the neuronal populations.

Despite the abundance of published studies carried out in primary CNS cultures, important aspects of neuronal differentiation *in vitro *remain ambiguous. The data reported here describe the long-term time course of maturation of isolated primary rat hippocampal cells in culture. We report that at 21 DIV, isolated hippocampal neurons completely switch from the expression of the intermediate type VI neurofilaments (nestin) to the synthesis of more advanced neurofilaments containing MAP-2. These data suggest that hippocampal cultures younger than 21 DIV do not resemble fully mature neurons as seen *in vivo *[11,6].

This culturing protocol uses a medium specifically formulated to promote neuronal survival and does not support glial proliferation [1]. However, it does not prevent the development of viable progenitor cells from differentiating into astrocytes during the development of the cell culture. Astrocytic development is sometimes viewed as a necessary event for the long-term survival of neuronal cells in culture [6,14]. We found that the prevalence of the neuronal phenotype (MAP-2 expressing cells) is maintained through all stages of the cell culture development. Cell populations in fully mature, long-term hippocampal cell cultures could include up to 20% GFAP-positive cells and relative numbers of astrocytes did not show further increase during the cell culture aging period. The gradual increase in GFAP expression and enrichment of the network of GFAP-positive cell processes, may be explained by slow (in comparison to neurons) maturation of existing astrocytes in B27-supplemented Neurobasal medium, which is not optimized to support astrocytic growth [6,1]. Alternatively, the enhanced arborization and GFAP expression may be attributable to astrocytic activation adjunct to the cell culture aging [33,5,13]. As *in vivo*, primary long-term cell culture aging is associated with the progressive oxidative damage of hippocampal neurons [5] and astrocytes are known to respond to various physiological or noxious stimuli including oxidative stress with increased expression of GFAP [14]. However, the results of our current study show signs of progressive degeneration of both MAP-2 and GFAP-containing networks during the later stages in the long-term hippocampal cell cultures.

Our study adds to the literature that investigates the endogenous release of Aβ [34,17,16,15,22,26] and potential deposition of amyloid-like aggregates in the process of development and aging of isolated hippocampal cells [17]. Consistently, we have detected the expression of APP and the presence of intracellular Aβ 1-42 immunoreactivity in long-term hippocampal cultures. The longitudinal monitoring of extracellular Aβ 1-42 immunoreactivity in primary hippocampal cell culture from DIV 4 until DIV 60 has shown that young, actively differentiating hippocampal cells have very low levels of physiological secretion of amyloidogenic Aβ. Direct Aβ ELISA measurements for 7-15DIV cell cultures that we report are consistent with similar data, which can be found in recent publications [26]. Notably, our experiments show that the increase of extracellular Aβ coincided with the complete maturation of synaptodendritic networks in hippocampal cultures, which indicates a potential relationship between increasing neuronal activity and the secretion of Aβ. Indeed, several recent studies [15,22,23,34] suggested that the formation of Aβ monomers play a role in the regulation of synaptic transmission.

Amyloid-like protein aggregates are thought to disrupt normal neuronal functioning by irreversible spontaneous insertion into cell membranes. The existing evidence suggests that the misfolded and aggregated proteins are essential elements in the vast majority of age-related neurodegenerative diseases [19]. Therefore, we used the Congo Red staining to determine whether the changes in extracellular levels of Aβ 1-42 were associated with the deposition of β-amyloids in long-term hippocampal cell cultures. The presence of Congo Red-positive aggregates associated with the network of processes and cell bodies of hippocampal cells appeared to mark the beginning of the period of decay in the long-term cell culture. Congo Red stained aggregates were consistently observed in long-term cultures after 30 DIV when the levels of extracellular Aβ 1-42 grew higher than 100 nM. The appearance of cell-associated amyloid-like aggregates became more frequent as the cell cultures continued to age and correlated well with morphological signs of deterioration of dendritic networks. Intriguingly, the cell culture ages when the presence of β-amyloidal aggregates were observed in the current study corresponded well with previously reported time course of protein oxidative damage in aging long-term hippocampal cell cultures [5,35,36].

The Aβ release and the following changes of the specific β-amyloid labeling preceded the decline of cell densities in aging hippocampal cultures. The pronounced increase in the number of cells exhibiting positive Congo Red staining of the somata in 60 DIV long-term cultures could be explained by the increased membrane permeability of decaying hippocampal cells which allowed Congo Red access to the intracellular compartment. Nevertheless, the overlap between the calcein AM and the Congo Red positive labeling indicated that, even in very old cultures, amyloid like aggregates could be found in the remaining alive hippocampal cells, which continue to maintain intact membrane integrity. On the other hand, the appearance of Congo Red fluorescence in dead (calcein AM-negative) cells is consistent with the suggestion that the presence of β-sheet structured protein aggregates may be a marker of neural cell injury and decay [37,38].

Studies of primary neuronal cultures beyond DIV 30 are infrequent [6,5,1]. Moreover, studies characterizing the development and senescence of neuronal cells *in vitro *without any treatment are difficult to identify in the literature. The current study not only joins the limited literature of long term cultures past DIV 30, we also characterized some important developmental milestones of note when using primary cell cultures. We found that neurons in primary culture undergo distinct developmental phases: differentiation, maturation, steady state, and senescence. Viability of these cell cultures was verified using immunoblotting of important structural proteins, immunoreactivity of important neuronal and glial markers, and differential interference contrast images to track visible damage on the cells.

Differentiation (DIV4-14) was characterized by the presence of nestin immunoreactivty and the prevalence of small cells with short processes and little to no branching in DIC images. Maturation (DIV 14-21) was classified as a time when the majority of nestin expressing cells began expressing MAP-2 (or GFAP), and a more defined network of neurites and larger, more defined cell bodies were present in DIC images. The steady state (DIV 21-35) was defined as the point in time when the neuronal cells in culture no longer displayed any noticeable changes. Senescence (DIV40-60) was defined as a decrease in total cell number, the presence of "beading" of the processes, a steady rise in Aβ production and an increase in aggregate formation.

## Conclusions

The phenomenon of cellular aging takes place in long-term primary neuronal cell cultures [13,4,5]. The present study found that endogenous cell senescence-associated amyloidogenesis *in vitro *occur in long-term hippocampal cell cultures. This outcome presents similar observations seen in the patterns of neuronal aging seen in animals, therefore, suggesting the possibility that long-term primary cell culture could serve as a model for particular aspects of aging, such as the study of neurodegenerative diseases. In sum, our results indicate that long-term primary cell cultures may be a useful instrument for designing and testing effective ways to delay neuronal senescence and other age related phenomenon.

## Methods

**Primary hippocampal cell cultures **were prepared from 18-day-old Sprague-Dawley rat fetuses. Procedures were carried out in accordance with the University of South Carolinas Institutional Animal Care and Use Committee. Rat hippocampi were dissected and incubated for 10 min in a solution of 2 mg/ml trypsin in Hank's balanced salt solution (HBSS) buffered with 10 mM HEPES (GIBCO Life Technologies, Paisley, Scotland). The tissue was then exposed for 2 min to soybean trypsin inhibitor (1 mg/ml in HBSS) and rinsed 3 times in HBSS. Cells were dissociated by trituration and distributed to glass bottom 35 mm and 96-well poly-L-lysine-coated plastic culture dishes (Costar, Cambridge, MA). Dishes were coated with a poly-L-lysine 48 hours prior to culturing. Initial plating densities were about 160-180 cells/mm^2^. At the time of plating, plates contained DMEM/F12 (GIBCO) supplemented with 100 mL/L fetal bovine serum (Sigma Chemicals, St. Louis, MO). After a 24-hr period, DMEM/F12 was replaced with an equal amount of Neurobasal medium supplemented with 2% v/v B-27, 2 mM GlutaMAX supplement and 0.5% w/v D-(1) glucose (all ingredients are from GIBCO). Neurobasal, B27-supplemented medium was used because it favors neuronal cell development and is not supportive for astrocytic proliferation. Cultures were maintained at 37°C in a 5% CO2/95% room air-humidified incubator at all times [5]. The long term maintenance of hippocampal cell cultures was carried out by replacing two thirds of the neurobasal medium with fresh medium after two weeks.

The morphology of hippocampal cells was examined at different time points (4, 7, 14, 21, 40, and 60 days in vitro (DIV) using differential interference contrast (DIC) microscopy as shown in Figure 1A. The fluorescent Hoescht staining of cell nuclei was used to monitor age-related changes in the cell density. The viability of hippocampal cells in long-term cultures was monitored by the fluorescent calcein AM/ethidium bromide cell labeling (Live/Dead cell viability kit, Invitrogen, Carlsbad, CA).

Immunofluorescent labeling with primary antibodies against nestin, MAP-2, and GFAP were used to assess the maturity of hippocampal neurons and detect cells with the neuronal astrocytic phenotype in long-term cultures of different ages (See Table 1 for antibodies used in this study). Nestin is a type VI intermediate filament protein transiently expressed by progenitor cells at the early stages of neurogenesis. Upon differentiation, nestin becomes down-regulated and replaced by neuron or astrocytic specific filaments, in this case MAP-2 and GFAP were used, respectively. Cells were fixed with an acetic acid/methanol solution for 5 minutes prior to blocking with a 10% horse serum solution. Secondary antibodies were Alexa Dye-conjugated host animal-specific IgG (Invitrogen). In all immunolabeling analyses the specificity of staining was confirmed by the exclusion of the subsequent primary antibody. The deposition of amyloid-like aggregates in the long-term hippocampal cell cultures was studied using the β-amyloid-binding Congo Red (Sigma Aldrich). Compared to other amyloid-binding dyes, such as thioflavin H or S, Congo Red exhibits very low non-specific binding and cannot be internalized by the cells, due to the presence of two hydrophilic sulfonic groups. Staining of non-fixed hippocampal cells with Congo Red and microscopic imaging of the results was carried out as previously described [39].

**Table 1:** Antibodies Used

Name	Method	Dilution	Animal Conjugate	Manufacturer
MAP-2	ICC/IF; WB	1:500; 1:1000	Rabbit Monoclonal	Santa Cruz

GFAP	ICC/IF;WB	1:5000	Chicken Monoclonal	Abcam

Glutamine Synthase	WB	1:10000	Mouse Monoclonal	Chemicon

Neuron Specific Enolase	WB	1:5000	Chicken Polyclonal	Aves

PSD-95	WB	1:1000	Goat Polyclonal	Abcam

β Actin	WB			

Aβ 1-42	WB; ICC/IF	1:500		Abcam

Nestin	ICC/IF	1:1000	Rabbit Polyclonal	Abcam

Anti-Rabbit IgG	WB	1:1500		Sigma

Anti-Mouse IgG	WB	1:1500		Sigma

Anti-Goat IgG	WB	1:1500		Abcam

Anti-Chicken IgG	WB	1:1500		Abcam

Total cell lysates prepared from hippocampal cells that had reached the ages of 14, 25, 35, and 45 DIV as previously described [40] were analyzed by Western blotting and using primary antibodies against neuron- or astrocyte-specific cytoskeletal (MAP-2 and GFAP) and cytosolic (neuron- specific enolase, NSE and glutamine synthetase, GS) marker proteins. In parallel Western blots of the hippocampal cell lysates were immunostained with antibodies against synaptic (PSD 95) and ubiquitous cytoskeletal proteins (β actin).

Direct ELISA measurements of the Aβ 1-42 immunoreactivity in cell-conditioned medium (CM) samples were performed using the rabbit polyclonal anti-Aβ 1-42 antibody. Serial dilutions of freshly prepared stocks of the synthetic rat Aβ 1-42 (0-2500 nM) (Anaspec, CA) with cell culture medium were used for calibration.

Immunofluorescent imaging was carried out under 20× magnification using the inverted fluorescent microscope (Nikon Eclipse TE2000-E) as previously described [4,40]. Immunofluorescent images, images of specific Congo Red binding, and DIC images were captured using a CCD camera. Merged images were produced and analyzed by the NIS Elements imaging software (Nikon). Hippocampal neurons (MAP-2 labeled cells) and astrocytes (GFAP labeled cells) were counted using the Object counting option of the NIS Elements imaging software package in 4 random fields of vision in cell cultures of different ages. For each field of vision total numbers of hippocampal cells were determined using the counting of Hoescht fluorescent stain of cell nuclei.

Statistical comparisons were made using ANOVA and planned comparisons were used to determine specific treatment effects. Significant differences were set at *P *< 0.05.

## Abbreviations

Aβ: Amyloid Beta; GFAP: Glial fibrillary acidic protein; APP: Amyloid precursor protein; GS: Glutamine synthase; CNS: Central Nervous System; MAP-2: Microtubule associated protein 2; DIC: Days *in vitro*; NSE: Neuron specific enolase; DIV: Differntial interference contrast; PSD 95: Post-synaptic density 95

## Authors' contributions

SJB assisted in carrying out immunohistochemistry, morphologically characterized cells, and drafted the manuscript. MVA carried out ELISA, and assisted in immunohistochemistry. MYA carried out Western Blot analysis, aided in manuscript preparation, and performed the statistical analysis. MVA and MYA conceived of the study, and participated in its design. CFM and RMB participated in the design and coordination of the experiments, as well as helping to draft the manuscript. All authors read and approved the final manuscript.
